# Use of Anaerobic Sludge Microbial Consortia in a Microbial Fuel Cell Biosensor for Biochemical Oxygen Demand Measurement

**DOI:** 10.3390/bios16080406

**Published:** 2026-07-26

**Authors:** Hebah Altaweel, Jamal Abu-Ashour, Borhan Aldeen Albiss, Bassim Abbassi

**Affiliations:** 1 Department of Civil Engineering, Jordan University of Science and Technology, Irbid 22110, Jordan; hhaltaweel21@eng.just.edu.jo (H.A.); jamals@just.edu.jo (J.A.-A.); 2 Nanotechnology Institute, Jordan University of Science and Technology, Irbid 22110, Jordan; baalbiss@just.edu.jo; 3 Department of Civil, Environmental, and Water Resources Engineering, University of Guelph, Guelph, ON N1G 2W1, Canada

**Keywords:** wastewater monitoring, biosensor, microbial fuel cell, electrogenic bacteria, BOD

## Abstract

Effective management of wastewater treatment plants often requires real-time measurements of Biochemical Oxygen Demand (BOD). Conventional methods for determining Biochemical Oxygen Demand (BOD) are often time-consuming, labor-intensive and prone to inaccuracies. Microbial Fuel Cells (MFCs) have emerged as a viable alternative technology for BOD measurement, offering real-time monitoring capability. However, challenges remain in its validity for testing different types of wastewater. This study developed a cost-effective dual-chamber MFC with graphite felt electrodes and a CMI-7000 membrane, inoculated with a microbial consortia grown from anaerobic sludge at optimal conditions (35 °C, pH 7, 1000 Ω external resistance). After one month of biofilm formation, the MFC produced 600 mV. Voltage outputs were measured at six BOD_5_ concentrations (36 to 583 mg/L) in synthetic wastewater, showing a strong linear correlation between BOD_5_ concentrations and voltage outputs. The MFC was also tested with five domestic wastewater samples with BOD_5_ values ranging between 81 and 405 mg/L. The output voltages were inserted into the derived voltage–BOD correlation to obtain BOD_5_ values within 2.5% to 11% of conventional laboratory results. These findings confirm the potential of MFC-based biosensors as an efficient and accurate tool for real-time wastewater monitoring.

## 1. Introduction

Biochemical oxygen demand (BOD) is a critical parameter in evaluating water quality and ensuring compliance with standards. It is defined as the amount of dissolved oxygen (DO) utilized by microorganisms in a water sample at a constant temperature of 20 °C over a specific time (typically five days) to degrade the organic matter present in the water [1,2]. There are many methods to measure BOD; the primary well-known accepted Standard Methods are the BOD_5_ test and the respirometric method [3]. The traditional BOD_5_, known for its precision, commences with the dilution of a sample in varying ratios using oxygen-saturated water and the introduction of nutrients and aerobic, heterotrophic microorganisms. The initial dissolved oxygen is measured before the sample is carefully placed in incubation in the dark at 20 °C, with a pH value between 7 and 8 for either 5 or 7 days, as per the preference of different countries. Subsequently, the DO content in the sample is quantified through either titration or electrochemically [4,5,6].

In the field of respirometry, manometry stands as one of the earliest techniques employed for analyzing the respiration of biological cultures, but the manometric technique was considered laborious and, as a result, was not widely utilized in wastewater applications. Notably, recent advancements in instrumentation and automation respirometers have successfully addressed limitations encountered in prior approaches. The respirometric method offers fast, accurate, and continuous measurement of microorganisms’ oxygen uptake throughout the incubation period. It has been employed to evaluate the degradation of chemicals over time and to assess the impact of toxic wastes on oxygen uptake [7]. Methods for estimating BOD have various limitations, notably a prolonged measurement period of at least 5 days and the susceptibility of used microorganisms to toxins and heavy metal ions, which can impede their respiration process and result in their demise [8,9,10]. Moreover, traditional methods lack sensitivity and precision, and the measurement procedure must be conducted within the first 24 h of sample acquisition [11]. This entails the development of new, convenient, and rapid methods that can be used for real-time monitoring of BOD [12].

Microbial fuel cell-based biosensor (MFC) has been considered a viable alternative technology to measure BOD. MFC has emerged as a promising technology for accurately, simply, and rapidly measuring BOD [13]. MFC is an electrochemical device that harnesses bacterial catalysis’s power to convert wastewater’s chemical energy into electricity [14]. Accordingly, the use of wastewater in the MFC makes it an environmentally friendly device as it also offers twofold benefits of waste management and bioelectricity generation [15]. Basically, MFC consists of the cathode and anode chamber divided by a proton exchange membrane. MFCs rely on biochemical reactions driven by microorganisms to generate electricity. The overall process involves the oxidation of organic substrates at the anode and the reduction of oxygen (or another electron acceptor) at the cathode. The summary reactions of an MFC are typically divided into two half-reactions:

Anodic Reaction (Oxidation): At the anode, microorganisms oxidize organic compounds (e.g., glucose, acetate, or other biodegradable matter), releasing electrons and protons. The electrons are transferred to the anode, while protons diffuse through a proton exchange membrane (if present) to the cathode.

Cathodic Reaction (Reduction): At the cathode, the electrons from the external circuit and the protons from the anode combine with an electron acceptor, often oxygen, to form water.

The following reactions illustrate typical electrode reactions using acetate as an example of the substrate, reaction (1), and reaction (2) for the anode and cathode, respectively [16]:

(1) $$Anode:{CH}_{3}{COO}^{-}\;+\;2H_{2}O\to \;{8e}^{-}+\;{2CO}_{2}+\;{7H}^{+}$$

(2) $$Cathod:2O_{2}+{8e}^{-}+{8H}^{+}\to \;{4H}_{2}O$$

The combined reactions (reaction (3)) result in the oxidation of organic matter and the reduction of oxygen, producing carbon dioxide, water, and electrical energy.

(3) $${CH}_{3}{COO}^{-}+{2O}_{2}+H^{+}\to {2CO}_{2}+{2H}_{2}O$$

When MFC uses organic matter as fuel, it produces current, and the concentration of this substance in the wastewater changes, which directly influences the resulting electricity output. Subsequently, MFC output fluctuations are monitored using simple devices. Remarkably, with thirty minutes to ten hours of response time, the BOD concentrations can be measured with high stability of the sensor’s performance [17,18].

At the core of biosensor functionality for BOD lies the crucial correlation between BOD concentrations and the measured charges or currents [19,20]. Many of these devices were developed on the basis of a dual-chamber MFC, where the anaerobic anodic chamber functions as the detection component utilizing organic matter in wastewater as the fuel for the MFC. The wastewater sample to be analyzed is introduced into the anodic chamber after the development of the mature anode biofilm, where the enrichment time can take place ranging from 3 to 8 weeks by the inoculation with activated sludge [18,21].

Several research efforts have focused on studying parameters that affect MFC-based BOD sensors. Ma et al. [22] studied how operational temperature affects the performance of MFC-based biosensors for measuring BOD in wastewater. A clear linear relationship has been observed between the biosensor’s output voltage and BOD values across different operating temperatures. With a similar intent, the effect of changes in environmental parameters, such as pH, on biosensor performance has been studied by Peixoto et al. [18], using wastewater that had a BOD_5_ concentration of 144 mg/L. The result showed that the highest current density recorded was 288 mA/m^2^ at pH 7.0. However, current densities were at their lowest, measuring 186 mA/m^2^ and 184 mA/m^2^, at pH levels of 6 and 8, respectively.

The microbial community is a critical factor that influences the performance of MFC-based sensor and requires careful monitoring to ensure optimal efficiency. Ideally, MFC-based sensor should incorporate microorganisms capable of metabolizing a wide variety of organic compounds. These sensors may utilize either pure microbial cultures with desirable characteristics such as broad substrate oxidation capabilities and resistance to environmental stressors, or microbial consortia, including engineered co-cultures and activated sludge cultures [23,24]. Studies have shown that the electrical output of MFC is greatly affected by the microbial communities present on the electrodes. The performance of MFCs can be significantly influenced by the type of microbial source used for inoculation during the startup phase [25]. Activated sludge, for instance, is a preferred microbial source for BOD detection, as it enables the bioreceptor to oxidize a broader range of organic substances. However, the biosensors may exhibit limited reproducibility over time because of the instability of the microbial consortium [23].

Recent research emphasizes that biofilm morphology, density, and microbial composition are pivotal in determining electrochemical performance. For instance, studies have shown that thicker, denser biofilms may impede effective electron diffusion, while sparse biofilms may result in suboptimal electron generation due to low bacterial colonization [26]. Therefore, achieving an optimal balance in biofilm structure is essential for maximizing MFC efficiency. Furthermore, operational parameters such as substrate type, pH, and electrode surface properties significantly influence the rate and quality of biofilm development [27].

Even the MFCs have shown a great result for BOD sensors but unfortunately, several limitations hinder widespread adoption. Many existing MFC-based biosensors rely on expensive materials, complex configurations, or pure microbial cultures, which restrict their practical implementation. Moreover, few studies have validated MFC sensors for different wastewater types over extended operational periods, limiting understanding of long-term stability and reproducibility. Furthermore, optimizing key operational parameters is critical to improve MFC performance and make them viable for practical use.

Unlike many previous MFC-based BOD biosensors that primarily focused on establishing BOD calibration relationships, the present study emphasizes practical applicability through the development of a low-cost dual-chamber MFC inoculated with an anaerobic sludge-derived mixed microbial consortium. In addition to evaluating BOD measurement performance, the study investigates key operational factors affecting sensor reliability, including different wastewater types, cathode operating conditions, and microbial community characteristics, thereby providing a more comprehensive assessment of the biosensor’s practical implementation.

This research addresses these challenges by pursuing the following objectives: (1) Construct an effective and low-cost microbial fuel cell for BOD measurement; (2) test the effectiveness and accuracy of the cell for measuring BOD in two types of wastewater (synthetic and domestic wastewater); (3) study the effect of cathode chamber pH on the MFC performance. Study the effect of catholyte type using tap water and deionized (DI) water on the MFC performance; and (4) characterize the microbial community within the anodic biofilm to identify dominant bacterial species contributing to electron transfer and evaluate their potential role in sensor stability and performance.

## 2. Materials and Methods

To assess the effectiveness of microbial fuel cells for biochemical oxygen demand detection and to evaluate system reproducibility under varying conditions, a series of controlled experiments were carried out. The experimental setup was designed to simulate both synthetic and domestic wastewater environments, providing a comprehensive understanding of MFC behavior across different influent types and operating parameters.

### 2.1. Synthetic Wastewater Composition

A solution comprising crucial components was prepared in the lab and utilized as synthetic wastewater. Its composition include glucose (1000 mg/L) the primary organic carbon source, 200 mg/L NH_4_Cl, 52.7 mg/L KH_2_PO_4_, 107 mg/L K_2_HPO_4_, 100 mg/L MgSO_4_·7H_2_O, 0.5 mg/L FeCl_3_, and 7.5 mg/L CaCl_2_ [28]. Collectively, these nutrients and trace nutrients ensured that the microorganisms had access to all the necessary elements to optimize their activity in the microbial fuel cell. The cleaning solution utilized in the anode chamber comprises all constituents found in the synthetic wastewater, except for glucose.

### 2.2. MFC System Configuration

**Figure 1:**
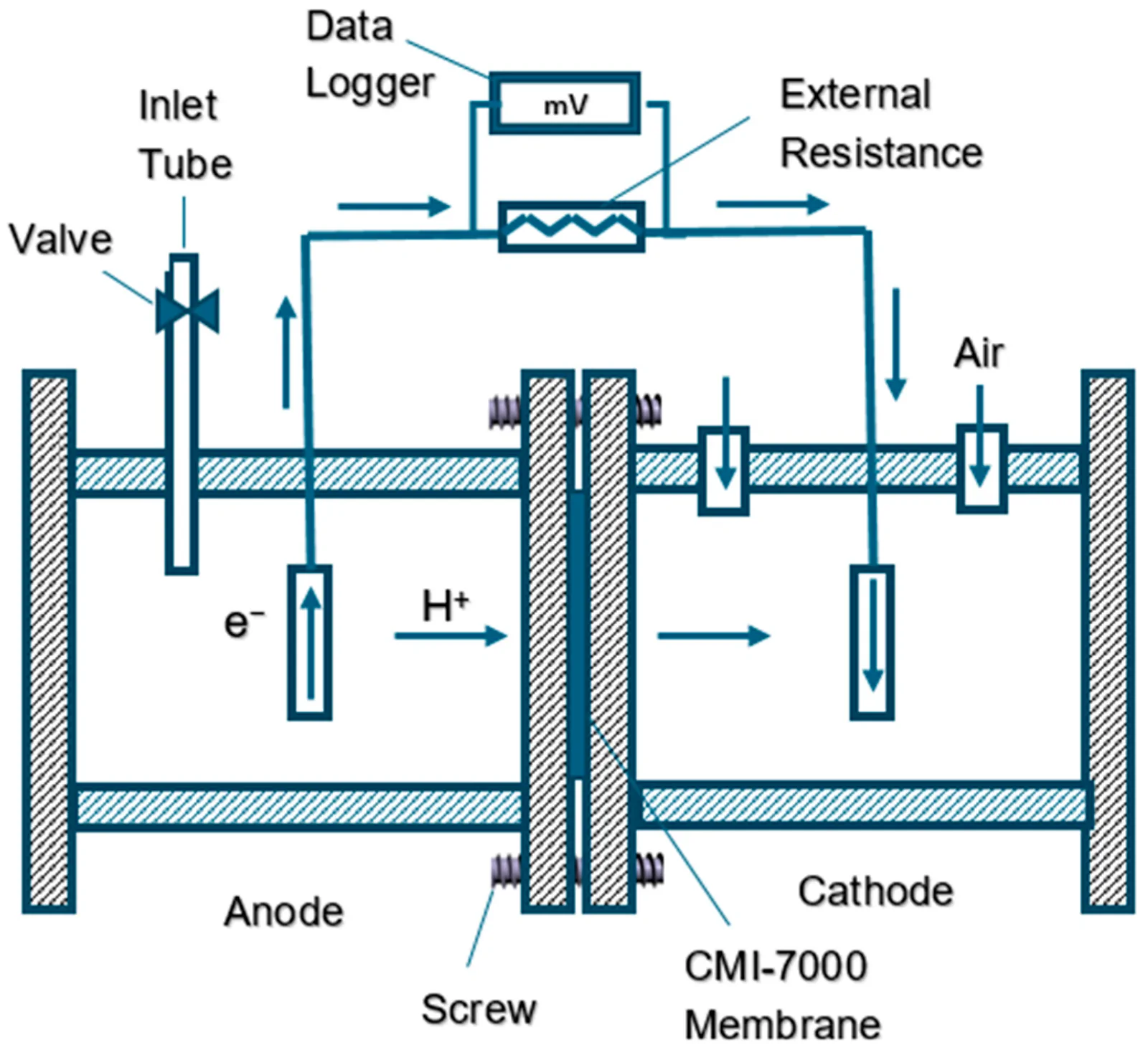
MFC system configuration.

The MFC biosensor’s architecture consisted of a conventional double-compartment design in a rectangular shape of (80 × 80 × 40 mm) and an inner diameter of 40 mm. The anode and cathode reactors were constructed of high-quality and durable acrylic, with a working volume of 100 mL each (Figure 1). The anode and cathode utilized Graphite Felt, with a thickness of 3.0 mm and 10.4 cm^2^ surface area, sourced from China. A proton exchange membrane CMI 7000 was employed to separate the two compartments. The electrodes were connected via a 1000 Ω external resistor using copper and stainless-steel wires. The external resistance of 1000 Ω was selected based on previous studies reporting its suitability for MFC operation and BOD sensing applications and that MFCs with higher external resistance would have a faster startup process.

### 2.3. Startup of the MFC

Anaerobic sludge from the Wadi Al-Shalala wastewater treatment plant in Irbid, Jordan was used for MFC enrichment and inoculation, serving as a source of electrochemically active bacteria. The sludge was cultivated on four types of agar media: red (blood agar), brown (chocolate agar), pink (MacConkey agar), and white (nutrient agar), and growth media under anaerobic conditions (candle jar method). These media were selected to enrich different microbial populations and obtain a diverse microbial consortium for MFC inoculation. At 35 °C, after 24 h of incubation, the enriched culture was mixed with synthetic wastewater (BOD 730 mg/L), with 20% of the total volume being bacterial culture. This mixture was injected into the anode chamber under a 1000 Ω external resistance, with nitrogen flushing to ensure anaerobic conditions. The cathode chamber, filled with DI water, remained aerobic, with oxygen supplied by an air pump. The mixture was replaced every other day until a sufficient population of microorganisms was enriched in the anode, and the MFC system reached a stable operating state, with the current pattern showing minimal variation.

### 2.4. BOD Detection and MFC Operation

This study tested DI water and tap water as cathode solutions with oxygen as the electron acceptor. Before BOD detection, the anode chamber was cleaned three times to ensure consistent initial conditions. Synthetic wastewater with BOD concentrations of 36–730 mg/L was introduced into the anode chamber. The system was operated under controlled conditions: 1000 Ω external resistance, 35 °C temperature (maintained by a water bath), and pH 7 for both chambers, with solutions replaced as needed to maintain stability.

The electrochemical performance was assessed by recording cell voltage using a data logger at one-minute intervals, and the steady-state time was determined from injection to peak stable output. Triplicate measurements were conducted for each sample, and a linear regression analysis was performed on wastewater samples to establish a standard curve for BOD detection.

### 2.5. Validation of BOD Detection

Five wastewater samples from the Jordan University of Science and Technology wastewater treatment plant were tested to validate the BOD detection curve. Each sample was injected into the MFC, and the resulting voltage output was recorded. BOD values were also determined using conventional methods for comparison. The MFC voltage output was compared with the established correlation curve, confirming the accuracy and reliability of the MFC-based BOD detection method. This validation demonstrated that the correlation curve accurately reflected the BOD levels in domestic wastewater samples.

### 2.6. Molecular and Structural Analysis of Bacterial Community

Understanding the microbial community and biofilm structure is crucial for optimizing MFC performance, as the activity of electrochemically active bacteria directly influences power generation and BOD detection. DNA was extracted using an in-house phenol-chloroform method, and bacterial identification was performed by amplifying the 16S rRNA gene with PCR (primers 27F and 1492R). The amplified products were sequenced by Macrogen (Macrogen, Inc. Seoul, South Korea) and analyzed using BLAST+2.8.1 for species identification. The biofilm structure on the anode was examined using a scanning electron microscope (SEM) (Quanta FEG 450, FEI, Hillsborom Oregonm USA). A dried section of graphite felt was mounted on aluminum stubs, coated with gold (Quorum Q 150R, Quorum Technologies, Sussex, UK), and analyzed under high vacuum.

## 3. Result and Discussion

### 3.1. Startup and Enrichment of MFC System

**Figure 2:**
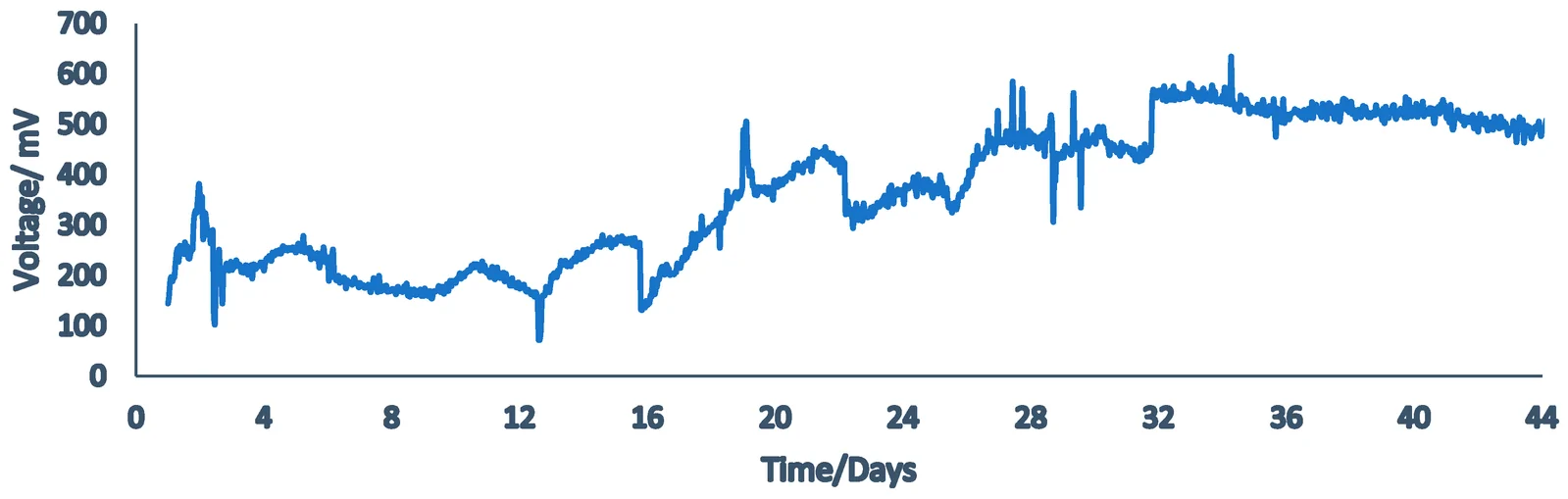
Variation in MFC output voltage over time during enrichment phases.

The MFC enrichment began with inoculation using a mixture of activated microorganisms and synthetic wastewater (BOD 730 mg/L) under batch mode (48 h feed cycles) at 35 °C, pH 7, and 1000 Ω external resistance. An initial voltage of 144 mV rose to 380 mV by the end of the first day. In subsequent cycles, only synthetic wastewater was supplied. The voltage increased steadily after each batch, peaking before declining as the substrate was consumed [11]. Over one month, the voltage rose from 144 to 600 mV, stabilizing at ~600 mV, indicating successful establishment of a stable microbial community on the anode. The voltage remained stable for ~48 h between feedings, defined as the stable period. The enrichment process lasted about one month, yielding a system suitable for detecting and measuring BOD in wastewater (Figure 2).

### 3.2. Effect of Cathode Conditions on MFC Voltage

**Figure 3:**
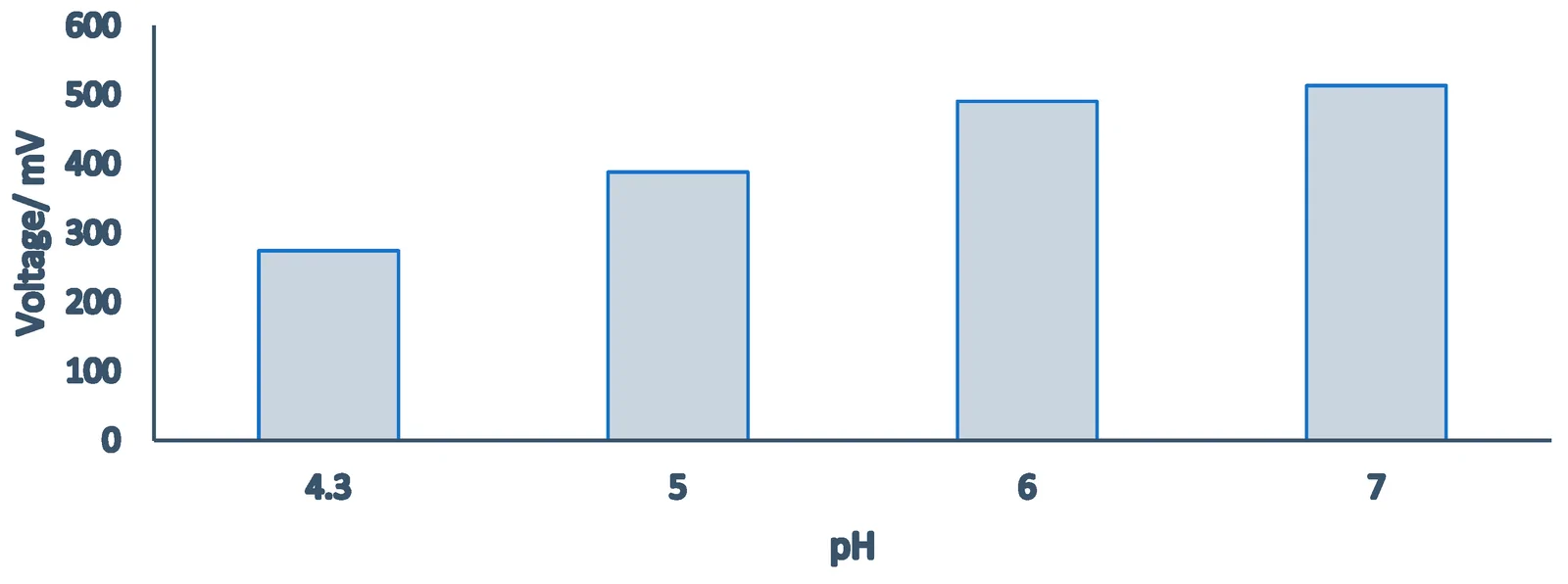
Effect of the pH in the cathode chamber on the MFC voltage output.

The pH is a critical factor significantly influencing power production in a MFC. Optimal pH levels are essential for maximizing microbial activity and facilitating efficient electron transfer processes. Typically, a pH close to neutral promotes microbial activity within an MFC [29]. Therefore, the anode chamber’s pH was maintained at around 7 for the whole experiment, as any deviation from this level can lead to a significant reduction in bacterial activity, ultimately hampering electron transfer and diminishing overall current production. However, during the MFC’s operation, the pH in the cathode chamber dropped significantly from 7 to 4.3 (Figure 3). This decrease was attributed to an imbalance between proton transfer from the anode chamber and proton consumption through the oxygen reduction reaction (ORR) at the cathode. Although H^+^ ions transferred from the anode chamber are consumed during cathodic reactions to form water, the rate of proton consumption at the cathode was insufficient to balance the rate of proton influx, resulting in proton accumulation and an increasingly acidic environment. Consequently, this hindered the oxygen reduction reaction, ultimately leading to a reduction in the overall power output [30]. To enhance cathodic oxygen reduction kinetics, ORR catalysts, such as platinum-based catalysts, are commonly used; however, their high cost may limit their application in low-cost MFC biosensors [30,31].

Deionized (DI) water and tap water were evaluated as catholyte solutions, using oxygen as the terminal electron acceptor. DI water, which had a higher dissolved oxygen (DO) concentration (6.2 mg/L), generated a greater voltage output (516 mV) compared to tap water, which had a lower DO level (3.7 mg/L) and produced only 338 mV. This performance difference can be attributed to the enhanced oxygen reduction reaction (ORR) kinetics at elevated DO levels, which facilitate a more positive cathode potential and consequently increase the overall cell voltage [30,32]. Higher DO availability at the cathode improves the electron-accepting capacity, thereby reducing the overpotential and improving energy conversion efficiency. These findings are consistent with previous research by Tao et al. [33], which reported a decline in maximum voltage output from 521 mV to 303 mV as the cathodic DO concentration decreased from 3.5 mg/L to 2.0 mg/L. The results underscore the importance of oxygen availability in optimizing MFC performance, particularly in relation to cathodic reactions. Overall, this demonstrates that dissolved oxygen concentration is a key operational parameter that should be carefully controlled to optimize electricity production and stability in microbial fuel cells.

### 3.3. Performances of MFC-Based BOD Biosensor

**Figure 4:**
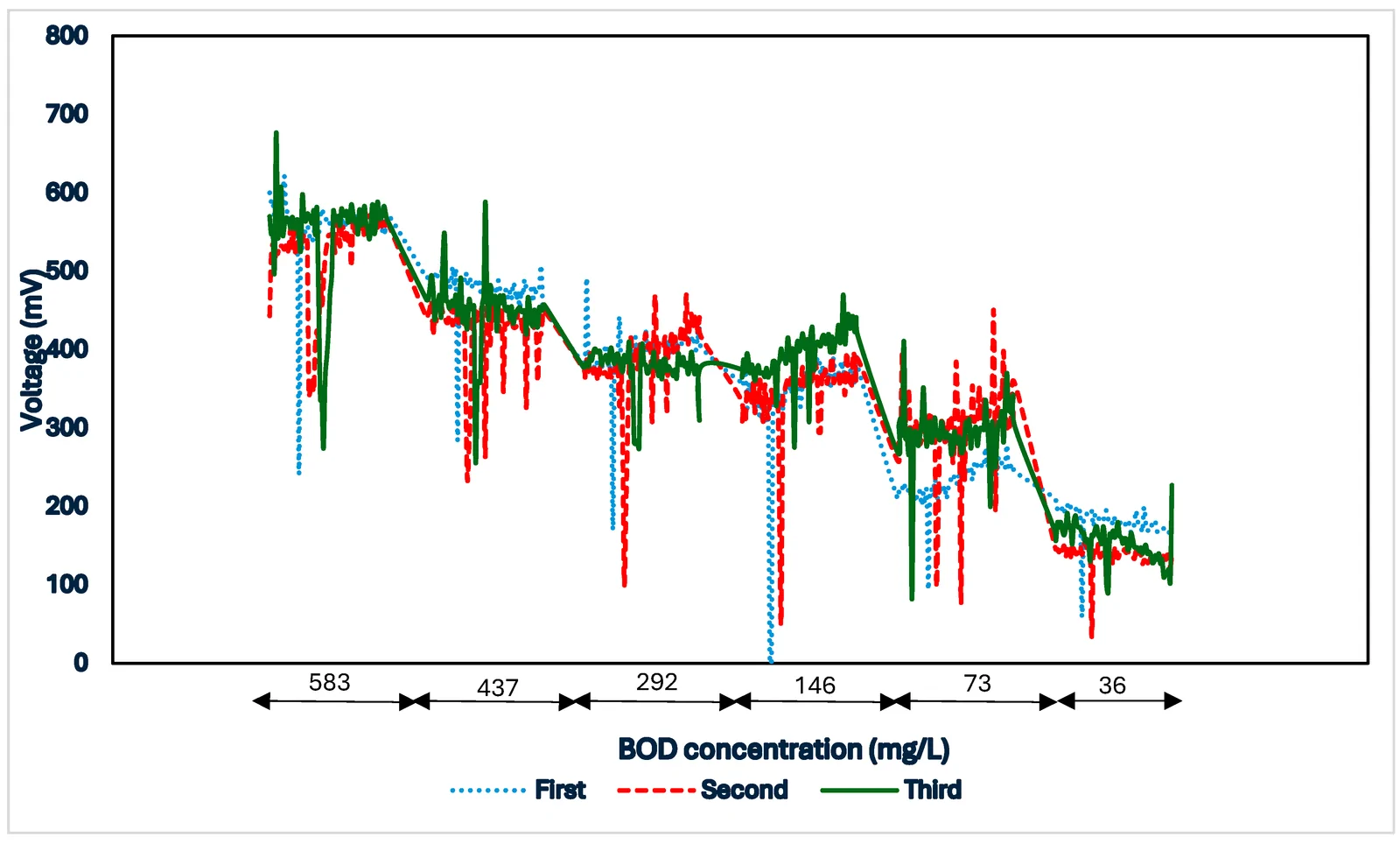
Consistency and accuracy of repeated concentration measurements.

**Figure 5:**
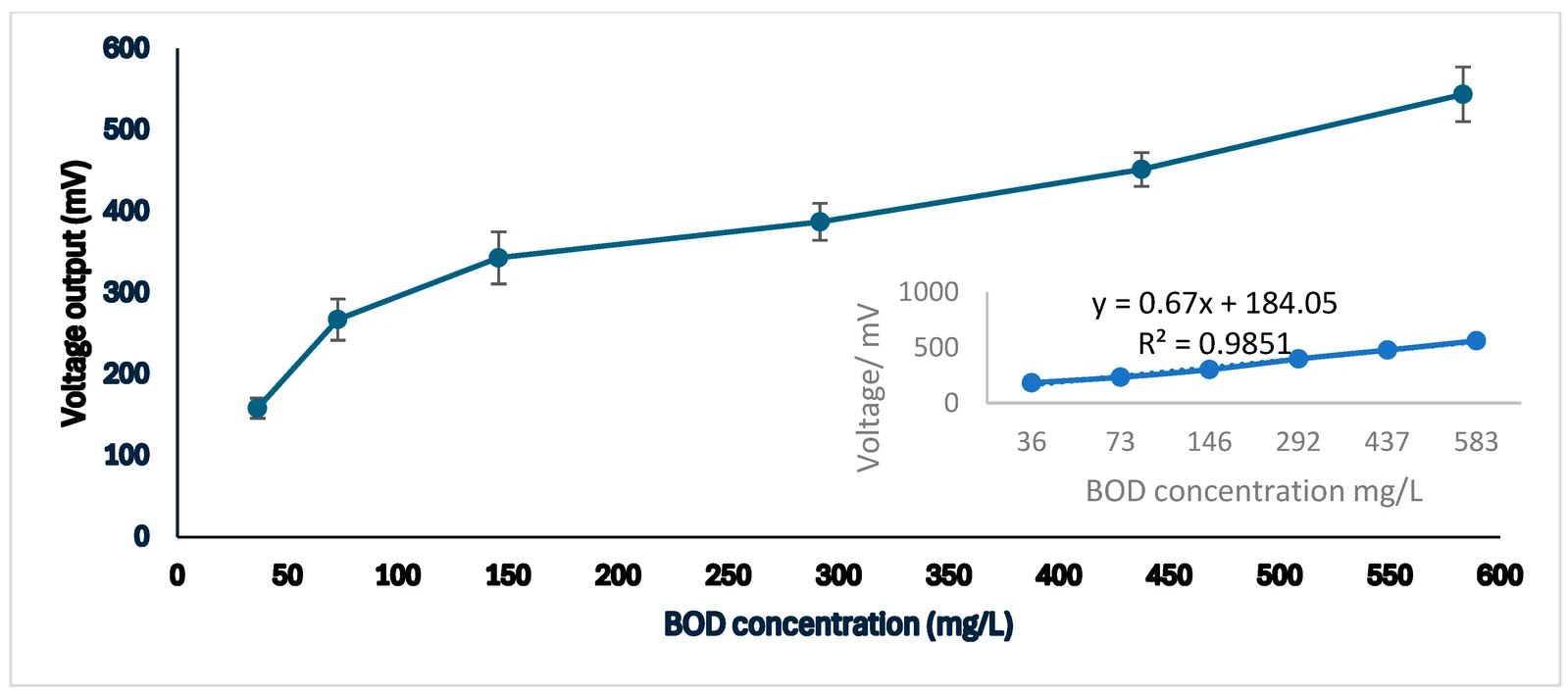
BOD calibration curve (BOD vs. voltage output).

After stabilization, the MFC was calibrated by correlating voltage with BOD concentrations ranging from 36 to 730 mg/L. A linear correlation was observed for BOD levels between 36 and 583 mg/L, with a correlation coefficient (R^2^) of 0.9851, indicating a good relationship between BOD concentration and the generated voltage signal. Beyond this concentration, the correlation diminished due to microbial saturation at higher BOD levels, and increased organic loading promoting methanogenic microorganisms, which impair electrogenic bacteria activity [34,35]. For each concentration (36, 73, 146, 292, 437, and 583 mg/L), three measurements were taken to ensure consistency. Figure 4 shows the continuous voltage reading of the data logger for the three measurements, highlighting their consistency and how closely they align, further validating the accuracy of the results. Each concentration was tested for 2 days. The sudden drops in the voltage at each concentration period is attributed to addition of new fresh substrate to the anodic chamber, a process that entails pumping out the old substrate and replacing it with a new one. The average output from these measurements was used to establish the BOD-MFC performance correlation curve (Figure 5). Figure 4 also shows that lower output voltage is measured as the BOD concentration decreases.

The developed MFC biosensor exhibited a response time of approximately 20 min with wide BOD concentration Range (36–750 mg/L), which compares favorably with most previously reported MFC-based BOD biosensors. reported response times generally range from 30 min to several hours or even days, depending on the microbial inoculum, electrode materials, and operating conditions. Yang et al. [36] obtained approximately 132 min of response time after introducing a BOD concentration of 200 mg/L. Hong Kim et al. [37] reported 300–600 min of response time for a 2.5–206 mg/L concentration, and 360–720 min with 8–240 mg/L concentration by Hsieh & Chung [38].

Compared with the conventional BOD_5_ method, which requires a 5-day incubation period, the developed MFC biosensor reduced the detection time to approximately 20 min, demonstrating its potential for rapid BOD monitoring.

### 3.4. Validation of MFC Performance

To validate the MFC biosensor, domestic wastewater was tested in addition to four dilutions (1:1, 1:2, 1:3, and 1:4), yielding five samples. The BOD_5_ in triplicate samples at each concentration were measured using the traditional 5-day BOD test, resulting in values of 405, 202, 135, 101, and 81 mg/L, respectively. Triplicate samples were also tested in the MFC, and the results are shown in Table 1. As clearly seen, the MFC results exhibited a strong correlation with the conventional BOD_5_ method, where the sensor’s BOD values were slightly lower, likely due to membrane pollution limiting proton flux and reducing current output.

Although characterization of membrane fouling was investigated in this study, membrane resistance can lower MFC electric output. An investigations involving membrane characterization to confirm this mechanism is required.

### 3.5. The Stability of the MFC Biosensor

**Figure 6:**
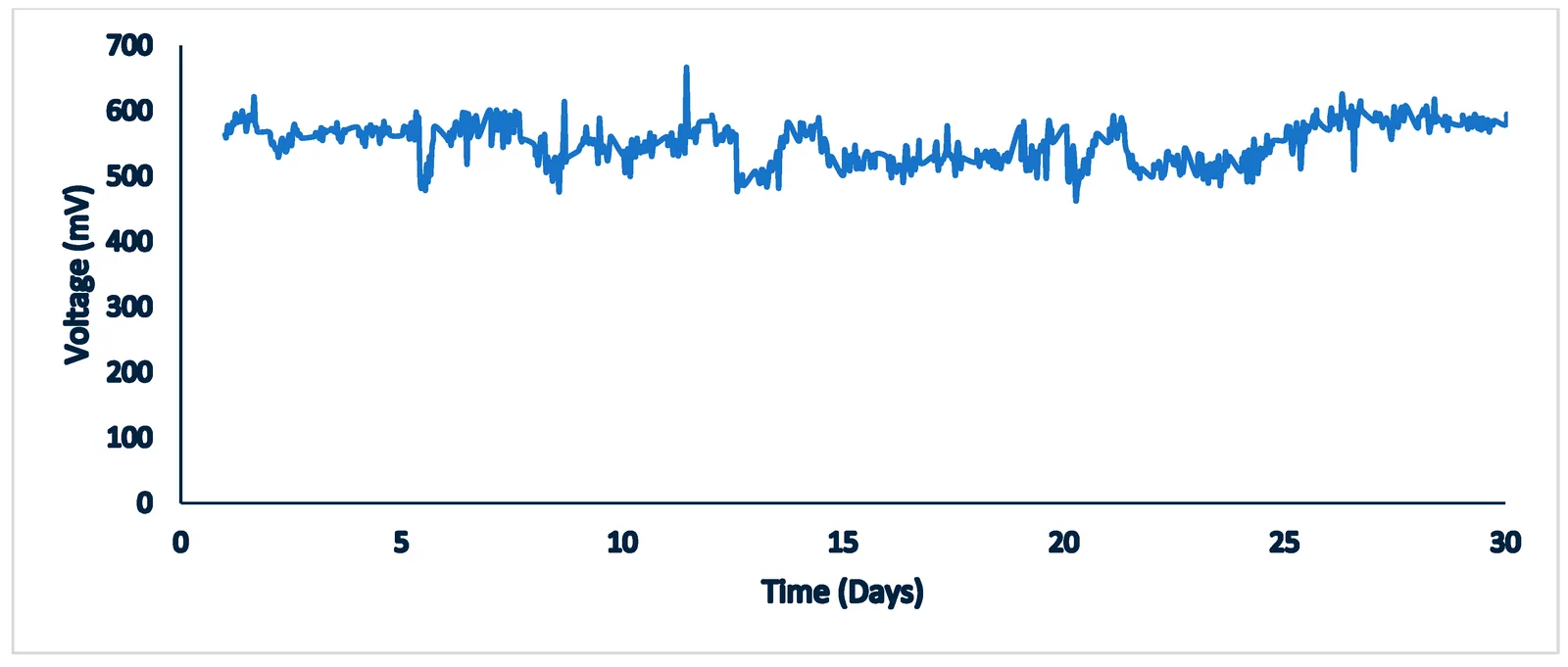
The stability of MFC through time.

Consistent performance throughout the desired operational period is crucial for ensuring the reliability of a biosensor system. To evaluate the stability of the microbial fuel cell biosensor, a protocol involving the introduction of 583 mg/L BOD for 30 days was implemented. This period is consistent with operational stability periods reported in previous biosensor studies ranging several days to approximately one month [38,39]. Figure 6 shows that the voltage output demonstrated consistent levels throughout the testing phase, yielding an average voltage of 551.71mV with a standard deviation of ±5.54%. Notably, our findings align with those of separate studies, where similar standard deviations of ±6.7% [39] and ±2.9% [38], were reported.

### 3.6. Biofilm Growth and Identification

**Figure 7:**
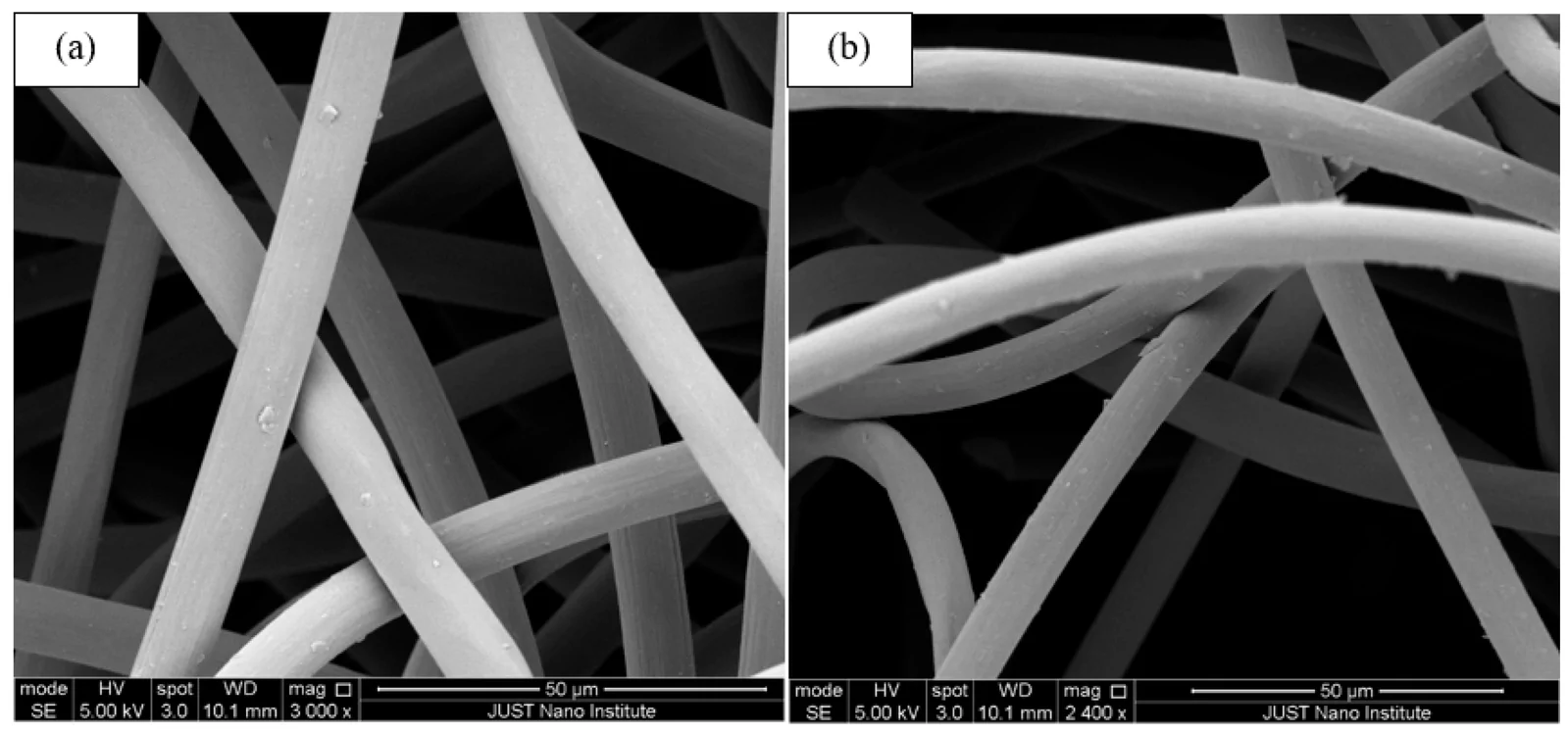

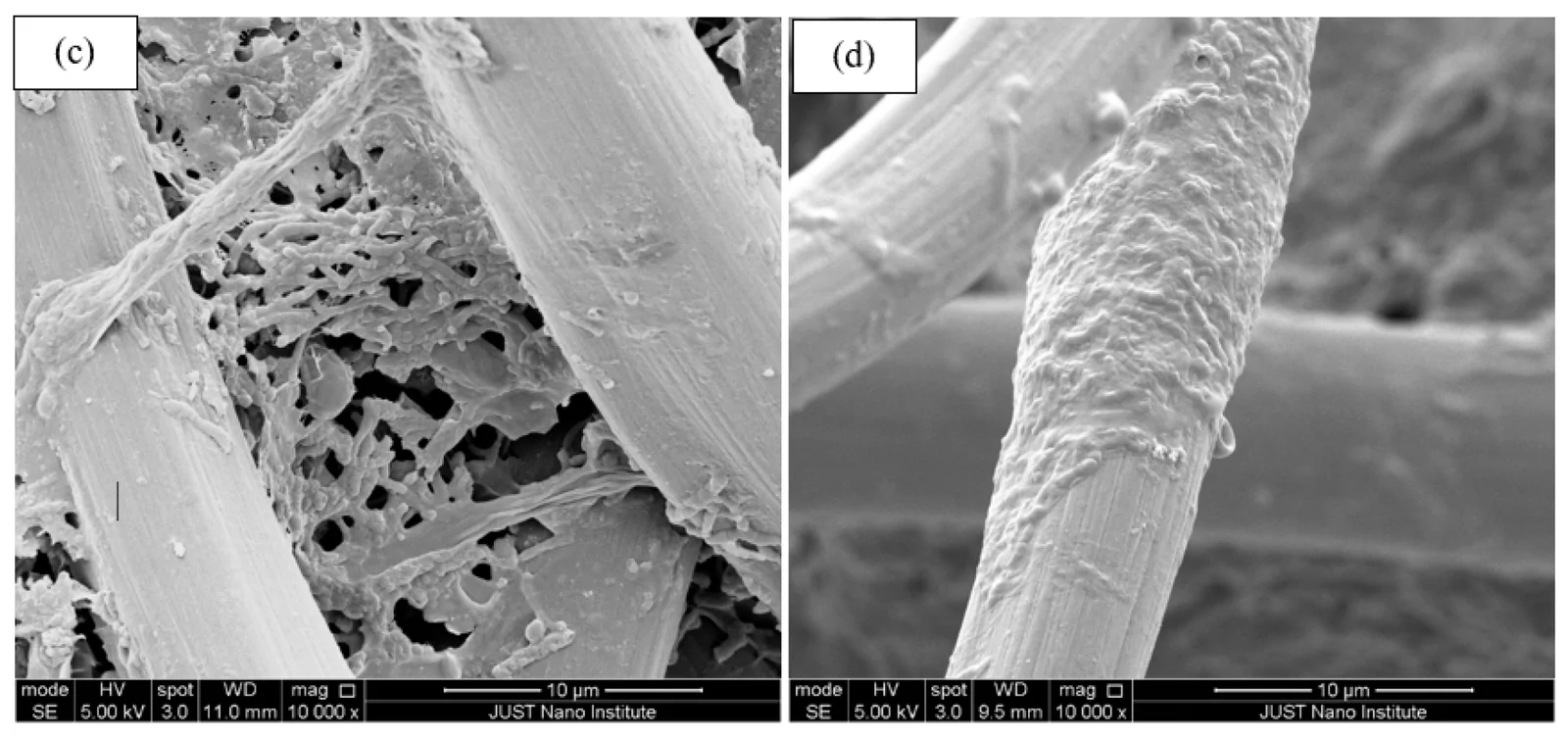
SEM images on graphite felt before and after biofilm formation ((**a**,**b**): clean graphite felt, (**c**,**d**): biofilms formation on the surface of the graphite felt anode).

The development of biofilm on the anode surface is a fundamental component influencing the performance of MFC biosensors. Biofilms, complex, structured communities of microorganisms, serve as biocatalysts that facilitate extracellular electron transfer from metabolic processes to the anode surface [29]. Their presence directly affects electron transfer kinetics, internal resistance, and overall power output of the system, making them critical to both energy generation and BOD detection. A well-established and stable biofilm enhances the MFC’s sensitivity and reliability, especially in long-term applications [40]. In this study, scanning electron microscopy (SEM) was employed to visualize and compare the structural characteristics of the anode electrode before and after biofilm development. As seen in Figure 7a,b, the pristine graphite felt electrode exhibited a relatively smooth and clean surface, indicative of its unused state. In contrast, Figure 7c,d display a visibly dense and heterogeneous layer of microbial cells adhered to the anode surface after operation, confirming successful biofilm colonization. The bacterial cells were observed to be embedded within a matrix of extracellular polymeric substances, a typical indicator of mature biofilm architecture.

These observations support the role of biofilm formation in enabling efficient electron transfer and validate the effectiveness of using anaerobic sludge as a microbial inoculum. The complex and robust microbial community that develops on the anode enhances the system’s capacity for sustained electron flow and consistent BOD detection.

The sequencing results obtained from 16S rRNA amplification and subsequent analysis of the enriched cultures obtained from the anaerobic sludge revealed several dominant strains closely related to known electroactive or metabolically versatile bacteria. Among the identified taxa, Shigella sp. strain PGM791 showed a 93.79% sequence identity with 98% query coverage (GenBank accession no. OQ876241.1). While Shigella is primarily known for its pathogenicity, recent studies have suggested that certain strains may exhibit exoelectrogenic activity under anaerobic or stress-induced conditions, particularly when part of a mixed microbial consortium [30]. This suggests that even traditionally non-electroactive or opportunistic bacteria can participate in extracellular electron transfer when environmental conditions are favorable.

A second major strain identified was a sulfide-oxidizing bacterium N9-1, with a 98.29% identity match, 99% query coverage (GenBank accession no. AF393509.1). Sulfide-oxidizing bacteria are well-documented contributors to current generation in microbial electrochemical systems, due to their ability to oxidize reduced sulfur compounds and transfer electrons to external solid-state acceptors like an-odes. Their presence in the biofilm implies an active role in enhancing the electrochemical activity of the MFC, particularly in environments where sulfur cycling is involved or where sulfide is present as a byproduct of organic matter degradation. Also identified was Bacterium CSR-57, with 98.71% sequence identity and 99% query coverage (GenBank accession no. KJ018060.1), which, while less characterized in terms of direct electron transfer capabilities, may play a supporting role in the microbial community. Such organisms can contribute to substrate breakdown, produce metabolic intermediates that fuel exoelectrogenic species, or enhance the structural stability of the biofilm matrix.

These findings highlight that both canonical electroactive species and non-traditional contributors can coexist within the anode biofilm and collectively support MFC performance. The functional cooperation among these microorganisms, whether through direct electron transfer, cross-feeding, or structural support, demonstrates the ecological complexity of bioelectrochemical systems [30].

## 4. Conclusions

This study developed and optimized a dual-chamber MFC as a biosensor for rapid BOD detection in wastewater. The system, using graphite felt electrodes and a CMI-7000 membrane, was inoculated with activated microorganisms and fed synthetic wastewater (BOD 730 mg/L) in 48 h cycles. After one month, a stable voltage of 600 mV was achieved, indicating successful biofilm formation on the anode. Operating conditions of 35 °C and pH 7 were maintained for optimal microbial activity. Catholyte pH was critical, as a drop to 4.3 reduced ORR efficiency, impacting voltage output. Experiments showed that DI water, due to its higher DO content, produced higher voltage than tap water. The MFC demonstrated linear voltage–BOD correlation with reliable accuracy up to 583 mg/L of BOD concentration (R^2^ = 0.9141), beyond which bacterial saturation led to a decline in performance. Domestic wastewater analysis (405, 202, 135, 101, and 81 mg/L) using MFC showed 2.5–11.11% differences compared to traditional 5-day BOD tests, validating the MFC’s accuracy. Over 30 days of operation at 583 mg/L, the MFC demonstrated consistent voltage output throughout the testing phase, with an average voltage of 551.71 mV and a standard deviation of ±5.54%.

A 16S rRNA sequencing identified a diverse microbial community, including *Shigella* sp., sulfide-oxidizing bacteria, and *Bacterium CSR-57*, essential for maintaining biofilm function. For practical applications, anaerobic sludges are considered a good candidate for inoculating an MFC. They are generally preferred as they are easily obtained from wastewater treatment plants, responsive to different substrates, and contain highly diverse electrochemically active strains of bacteria. Moreover, they are available in large quantities and can tolerate environmental fluctuations. Also, studies have shown that the highest open-circuit voltage and power density were obtained from wastewater-inoculated MFCs, whereas soil- and sediment-inoculated MFCs exhibited lower outputs.

This research presents a promising, reliable, rapid, and practical alternative for BOD monitoring, without compromising analytical reliability as evidenced by the low deviation (2.5–11.11%) from the conventional BOD_5_ method during domestic wastewater validation. This system presents an improvement to the existing biosensor systems by employing a simple dual-chamber configuration with anaerobic sludge as a mixed microbial inoculum and graphite felt electrodes, reducing operational complexity while maintaining stable performance. These findings demonstrate the potential of the developed MFC as a simple, self-powered platform for rapid and online BOD monitoring, although further validation under long-term field conditions is required before practical implementation.

Overall, this study demonstrates the effectiveness of the developed MFC in providing timely BOD_5_ measurement which is vital for effective wastewater management. The study recommends further testing of the MFC for BOD_5_ measurements in other types of wastewaters such as industrial effluents. The study also recommends testing the stability of the MFC for several months to assess the effects of membrane fouling and electrode deterioration on performance of the MFC.

## Figures and Tables

**Table 1:** Comparison of BOD_5_ and sensor BOD for domestic wastewater.

Sample	BOD Sensor (mg/L)	BOD_5_ (mg/L)	Difference (%)
1	375	405	7.5
2	180	202	11.1
3	132	135	2.5
4	93	101	8.1
5	72	81	10.7

## Data Availability

All data that support the findings of this study are included.
